# GLP-1 Receptor Agonists: Encouraging Signals for Treating Alcohol Use Disorder

**DOI:** 10.1007/s11606-025-09498-3

**Published:** 2025-04-24

**Authors:** Marlene C. Lira, Eileen Barrett, M. Justin Coffey

**Affiliations:** 1 Workit Health, Albuquerque, NM USA; 2 DrPH Program, Health Policy, Johns Hopkins Bloomberg School of Public Health, Baltimore, MD USA; 3 Workit Health, Ann Arbor, MI USA; 4 Geisinger Commonwealth School of Medicine, Scranton, PA USA

**Keywords:** GLP-1, Alcohol use disorder, Addiction

## Abstract

Excessive alcohol use is a leading cause of preventable death in the USA, and the number of annual deaths from alcohol more than doubles the number from drug overdose. As newer glucagon-like peptide-1 (GLP-1) receptor agonists like semaglutide have increased in use, anecdotal reports suggest they may reduce craving for alcohol and other addictive substances. Given this increased attention, this perspective reviews findings from one trial and three recent retrospective studies assessing relationships between GLP-1 agonists and alcohol-related health outcomes. The one published GLP-1 trial for alcohol use to date resulted in null findings overall, but significant reductions in heavy drinking days and alcohol use among a subgroup of participants with obesity. The three retrospective analyses found associations between GLP-1 agonists and multiple outcomes including incident and recurrent alcohol use disorder, alcohol intoxication among individuals with alcohol use disorder, and hospitalizations related to alcohol use, substance use, and somatic health conditions. Considerations for the use of GLP-1 agonists in the context of alcohol use disorder are discussed, as well as outstanding questions and limitations with the current evidence. Additional clinical trials are needed to provide definitive answers, and to open the pathway for FDA approval and payer coverage.

Glucagon-like peptide- 1 (GLP-1) receptor agonists have surged in use for diabetes, obesity, and overweight since 2022, and, as of 2024, 12% of US adults reported using one of these medications.^1^ In tandem, anecdotal evidence has grown about the potential of these medications to reduce craving for alcohol and other addictive substances.^2^ We describe the scientific evidence on these medications for reducing alcohol consumption among humans and considerations for their use.

Over 10% of the US population ages 12 years and older is living with alcohol use disorder (AUD).^3^ Excessive alcohol use is a leading cause of preventable death in the USA, and the number of annual deaths from alcohol more than doubles the number from drug overdose.^4^ Although there are three Food and Drug Administration (FDA)-approved and multiple off-label medications used to treat AUD, both first-line medications (naltrexone and acamprosate) and second-line medications (topiramate, gabapentin, baclofen) have had limited uptake; less than 2% of individuals with AUD receive medication.^5^ This is due to multiple factors ranging from stigma and low public awareness of medications to the inadequate supply of addiction-trained clinicians and inadequate supports for non-addiction-trained clinicians. Furthermore, the combination of low alcohol taxes with the normalization of excessive alcohol use in American society may make it challenging for individuals to recognize harmful alcohol consumption patterns and seek care. Even with optimal prescribing patterns, however, the most effective of the available AUD medications are still less effective in treating AUD than the most effective medications available for opioid use disorder (e.g., buprenorphine and methadone).^6,7^ Identifying or developing additional pharmacotherapies for AUD is critical. The widespread interest in GLP-1 and gastric inhibitory polypeptide (GIP) agonists and their integration into public discourse may therefore present an opportunity to expand AUD treatment.

To date, a handful of studies have been published on the use of GLP-1 agonists and alcohol use in humans, including results from one randomized, placebo-controlled trial. The trial tested exenatide, an early GLP-1 agonist with reduced potency in comparison to newer medications such as semaglutide and tirzepatide.^8^ Exenatide did not significantly reduce the number of heavy drinking days compared to placebo in the overall analysis. However, in a subgroup analysis of participants with obesity, exenatide significantly reduced the number of heavy drinking days and total alcohol consumption compared to placebo.

More recently, three retrospective cohort analyses have demonstrated consistent and promising findings related to associations between newer GLP-1 s and adverse alcohol-related health outcomes. Wang et al. conducted a retrospective cohort study utilizing electronic health record data that examined relationships between semaglutide and incident and recurrent diagnoses of AUD.^9^ The authors stratified analyses by diagnoses of obesity and type II diabetes, and compared semaglutide to AUD medications naltrexone and topiramate as well as other non-GLP-1 obesity and diabetes medications. Among patients with obesity, those who were prescribed semaglutide had a 56% reduction in the risk of having a new diagnosis of AUD and a 75% reduction in the risk of having a recurrent diagnosis of AUD over time compared to those prescribed naltrexone or topiramate. This is striking given that naltrexone and topiramate are used to treat AUD. The study also found protections for incident and recurrent diagnoses of AUD among individuals with obesity when comparing semaglutide to non-GLP-1 obesity medications as well as among individuals with diabetes when comparing semaglutide to non-GLP-1 diabetes medications. Results for individuals with diabetes were consistent across obesity status, which is novel given the null finding of exenatide on alcohol consumption in the aforementioned trial.^8^ The study also found that protective effects were sustained within a subset of individuals who took medications for three years. This is important because previous studies have not assessed longer time periods.

A retrospective cohort analysis by Qeadan et al. utilized medical record data of over 800,000 adults with AUD from 136 health systems in the USA.^10^ In adjusted models, use of a GIP/GLP-1 was associated with a 50% reduction in the rate of alcohol intoxication. Similar to the study by Wang et al., these findings remained consistent in analyses stratified by diabetes and obesity diagnoses.

Lastly, Lähteenvuo et al. conducted an analysis using a complete census of Swedish adults with AUD.^11^ Semaglutide and liraglutide were associated with 36% and 28% reductions in the risk of AUD-related hospitalization over time, respectively. In contrast, use of any AUD medication (i.e., naltrexone, acamprosate, disulfiram) was only associated with a 2% reduction in the risk of AUD hospitalization. In addition, semaglutide and liraglutide were associated with reduced risks of hospitalizations from substance use disorder and somatic health conditions. Importantly, the authors also assessed whether GLP-1 medications were associated with hospitalizations for suicide and found no associations. Because individuals would have likely only been prescribed GLP-1 agonists for obesity/diabetes during this time period, this study again suggests that, among individuals with metabolic conditions and AUD, GLP-1 agonists may be more effective in reducing adverse alcohol-related outcomes than typical AUD medications. This study also examined relationships between exenatide and hospitalizations but the findings were not statistically significant, suggesting there may be differential effects by type of medication.

Finally, there have been encouraging signals in the literature regarding GLP-1 agonists and other health conditions related to excessive alcohol use. A retrospective cohort study using data from the Veterans Health Administration of individuals with metabolic dysfunction-associated steatotic liver disease and diabetes found that, compared to dipeptidyl peptidase 4 inhibitors, GLP-1 agonists were associated with lower risk of cirrhosis, cirrhosis complications, and mortality.^12^ In addition, a study using registry data from Sweden and Denmark found that GLP-1 agonists were not associated with increased risk of adverse mental health outcomes including suicide, nonfatal self-harm, or incident depression or anxiety.^13^

Given the limited efficacy of existing pharmacotherapies for AUD, clinicians often prescribe off-label medications for AUD. Clinicians could similarly consider prescribing GLP-1 agonists for AUD, even before randomized controlled trial results are published or FDA approval is obtained. However, major barriers to utilizing these medications are cost and availability. Given that AUD is not an approved indication, it is unlikely payers would cover them. It is possible that individuals could have the medications covered through other health conditions, but even then coverage is highly variable. Furthermore, as of early 2025, the high cost and limited availability of these medications are leading many consumers to compounded formulations, some of which may be ineffective or potentially harmful.^14,15^

These studies are promising, yet clinical trials are still needed to provide definitive answers, and to open the pathway for FDA approval and payer coverage. Although these retrospective studies suggest GLP-1 agonists may be effective in reducing incident and recurrent AUD, alcohol intoxication among individuals with AUD, and AUD-related hospitalizations, there may nevertheless be unmeasured differences between the groups that could fully be accounted for through a prospective, randomized controlled trial. There may be selection bias in that individuals with AUD and diabetes or obesity who seek treatment may be different than individuals who do not seek or obtain treatment. Similarly, missing or inaccurate data in claims datasets may confound these findings. Additionally, it is currently unclear whether GLP-1 medications may confer benefits among individuals without any metabolic conditions. As the study by Lähteenvuo et al. suggests, there may also be differences between GLP-1 agonists. Similarly, dosing for AUD may differ from dosing for diabetes/obesity, and differences between oral and injectable GLP-1 formulations have not been assessed. There may be previously unidentified risks with the use of these medications among individuals with AUD. Lastly, additional research is needed to understand how these medications could work in conjunction with existing medications for AUD as well as psychosocial interventions that could address the complex social and mental health needs that are prevalent among individuals with AUD. Until there are randomized controlled trials demonstrating both the safety and efficacy of GLP-1 agonists in treating AUD, we recommend clinicians first consider naltrexone or acamprosate, and in an evidence-informed manner consider the potential promise and risks of GLP-1 agonists.

## Data Availability

Not applicable.
